# Supplementary material for: PPAR Pan Agonist MHY2013 Alleviates Renal Fibrosis in a Mouse Model by Reducing Fibroblast Activation and Epithelial Inflammation
Source: Int J Mol Sci. 2023 Mar 2;24(5):4882. doi: 10.3390/ijms24054882 (PMC10002481; doi:10.3390/ijms24054882)
Supplement: Supplementary file 1 [file ijms-24-04882-s001.zip › ijms-2187175-supplementary.pdf]

Table S1. Information of Antibodies used in Western blotting

**Antibody**	**Company**	**Catalog number**
α-SMA	Santa Cruz	sc 32251
Col1a1	Santa Cruz	sc 293182
Col1a2	Santa Cruz	sc 393573
Vim	Cell signaling	#5741
p-SMAD2	Abcam	Ab184557
p-SMAD3	Abbkine	ABP50494
p65	Santa Cruz	sc 514451
p-p65	Santa Cruz	sc 136548
β-actin	Santa Cruz	sc 47778
ɑ-Tubulin	Santa Cruz	sc 5286

Table S2. Primer sequences for qPCR

**Mouse**		
Gene	*Forward (5'-3')*	*Reverse (3'-5')*
Havcr1	*GTG GAA GTA AAG GGG GTG GT*	*TGC CCC TTT AAG TTG TAC CG*
Timp2	*GCA TCA CCC AGA AGA AGA GC*	*GGG TCC TCG ATG TCA AGA AA*
Igfbp7	*ATG AAG GAG CTG GCT GTG TT*	*GGG ATG TGC AGG GAG TAG AG*
Spp1	*TCT GAT GAG ACC GTC ACT GC*	*AGG TCC TCA TCT GTG GCA TC*
Col1a2	*GAT GGC TGC TCC AAA AAG AC*	*CAA TGT CCA GAG GTG CAA TG*
Col3a1	*AGG ATC TGT CCT TTG CGA TG*	*TCT CCA AAT GGG ATC TCT GG*
VIM	*ATG CTT CTC TGG CAC GTC TT*	*AGC CAC GCT TTC ATA CTG CT*
Tnfα	*GCT TTC CGA ATT CAC TGG AG*	*TTG CAC CTC AGG GAA GAA TC*
Il1β	*GCT GCT TCC AAA CCT TTG AC*	*TTC TCC ACA GCC ACA ATG AG*
Ccl2	*CTG GAT CGG AAC CAA ATG AG*	*TGA GGT GGT TGT GGA AAA GG*
Emr1	*ACC ATC ACC TAT GGA CCC AA*	*CCC AGT CAT GGT CTC CAG TT*
GAPDH	*AAG GTC ATC CCA GAG CTG AA*	*CTG CTT CAC CAC CTT CTT GA*

Rat

Gene	***Forward (5'-3')***	***Reverse (3'-5')***
Ccl2	*GCCAACTCTCACTG AGCCA*	*GCATCTGGCTGAGACAGCAC*
Ccl5	*ATATGGCTCGGACACCACTC*	*CCACTTCTTCTCTGGGTTGG*
Cxcl1	*GCTGGGATTCACCTCAAGAA*	*TGGGGACACCTTTTAGCATC*
Col1a2	*TTG ACC CTA ACC AAG GAT GC*	*CAC CCC TTC TGC GTT GTA TT*
Acta2	*ACT GGG ACG ACA TGG AAA AG*	*CAT CTC CAG AGT CCA GCA CA*
Vim	*TGC ACG ATG AAG AGA TCC AG*	*TGG CAG CCA CAC TTT CAT AC*
GAPDH	*TGC TGG TGC TGA GTA TGT CG*	*AGT TGG TGG TGC AGG ATG C*
